# The Molecular Epidemiology and Evolution of Murray Valley Encephalitis Virus: Recent Emergence of Distinct Sub-lineages of the Dominant Genotype 1

**DOI:** 10.1371/journal.pntd.0004240

**Published:** 2015-11-24

**Authors:** David T. Williams, Sinéad M. Diviney, Aziz-ur-Rahman Niazi, Peter A. Durr, Beng Hooi Chua, Belinda Herring, Alyssa Pyke, Stephen L. Doggett, Cheryl A. Johansen, John S. Mackenzie

**Affiliations:** 1 CSIRO, Australian Animal Health Laboratory, Geelong, Victoria, Australia; 2 Faculty of Health Sciences, Curtin University, Perth, Western Australia, Australia; 3 Office of Research and Development, Curtin University, Perth, Western Australia, Australia; 4 Infectious Diseases and Immunology, University of Sydney, New South Wales, Australia; 5 Public Health Virology, Queensland Health Forensic and Scientific Services, Coopers Plains, Queensland, Australia; 6 Department of Medical Entomology, Westmead Hospital, University of Sydney and Institute for Clinical Pathology and Medical Research, New South Wales, Australia; 7 Arbovirus Surveillance and Research Laboratory, School of Pathology and Laboratory Medicine, University of Western Australia, Perth, Western Australia, Australia; University of Texas Medical Branch, UNITED STATES

## Abstract

**Background:**

Recent increased activity of the mosquito-borne Murray Valley encephalitis virus (MVEV) in Australia has renewed concerns regarding its potential to spread and cause disease.

**Methodology/Principal Findings:**

To better understand the genetic relationships between earlier and more recent circulating strains, patterns of virus movement, as well as the molecular basis of MVEV evolution, complete pre-membrane (prM) and Envelope (Env) genes were sequenced from sixty-six MVEV strains from different regions of the Australasian region, isolated over a sixty year period (1951–2011). Phylogenetic analyses indicated that, of the four recognized genotypes, only G1 and G2 are contemporary. G1 viruses were dominant over the sampling period and found across the known geographic range of MVEV. Two distinct sub-lineages of G1 were observed (1A and 1B). Although G1B strains have been isolated from across mainland Australia, Australian G1A strains have not been detected outside northwest Australia. Similarly, G2 is comprised of only Western Australian isolates from mosquitoes, suggesting G1B and G2 viruses have geographic or ecological restrictions. No evidence of recombination was found and a single amino acid substitution in the Env protein (S332G) was found to be under positive selection, while several others were found to be under directional evolution. Evolutionary analyses indicated that extant genotypes of MVEV began to diverge from a common ancestor approximately 200 years ago. G2 was the first genotype to diverge, followed by G3 and G4, and finally G1, from which subtypes G1A and G1B diverged between 1964 and 1994.

**Conclusions/Significance:**

The results of this study provides new insights into the genetic diversity and evolution of MVEV. The demonstration of co-circulation of all contemporary genetic lineages of MVEV in northwestern Australia, supports the contention that this region is the enzootic focus for this virus.

## Author Summary

Murray Valley encephalitis virus is the most significant cause of mosquito-borne encephalitis in humans in Australia, and can also cause neurological disease in horses. This study reports an expanded phylogenetic study of this virus and the first molecular evolutionary analysis. Of the four recognized genotypes of Murray Valley encephalitis virus, only two were found to be actively circulating (genotypes 1 and 2), and genotype 1 was dominant. Distinct genetic sub-lineages within genotype 1 were found to have recently emerged. Molecular clock analysis indicated that genotype 2 viruses are the oldest genetic lineage while genotype 1 viruses are the most recent to diverge. The co-circulation of distinct genetic lineages of this virus in northwestern Australia, comprising the oldest and youngest lineages, supports previous findings that MVEV circulates endemically in this region.

## Introduction

Murray Valley encephalitis virus (MVEV; genus *Flavivirus*, family *Flaviviridae*) is the most important cause of arboviral encephalitis in Australia. It is a member of the Japanese encephalitis (JE) serocomplex, along with other medically important flaviviruses such as JE virus (JEV) and West Nile virus (WNV), which also circulate in the Australasian region [1]. MVEV exists in zoonotic cycles between *Culex* species mosquitoes and water birds (reviewed in [2]). It is enzootic in northern parts of Australia, including the Kimberley region of Western Australia and northern areas of the Northern Territory, with frequent spillovers into the Pilbara and Gascoyne regions of Western Australia, and occasional spread to southern areas of the Northern Territory and northern Queensland [3–5]. MVEV very occasionally spreads into south-eastern Australia following periods of heavy and prolonged rainfall, where it has caused major epidemics in the last century, centred in the Murray Valley region [2,3,6]. Since the last nationwide epidemic of MVE in 1974, there have been approximately 127 human cases, with the majority occurring in Western Australia or the Northern Territory. Outside Australia, MVEV is believed to be endemic in Papua New Guinea (PNG), where human cases, serological evidence of infection, and virus isolations have been reported [7–9]. In addition to human disease, MVEV can cause fatal encephalitis in horses [10,11].

In recent years, MVEV has become increasingly active in south-eastern Australia, raising concerns that the virus may become established in enzootic transmission cycles in populous regions. Seroconversions to MVEV were found in sentinel chicken flocks in New South Wales in 2001 and 2003 [12], and in 2008 the first human case since the 1974 epidemic occurred in this state along with sentinel chicken seroconversions [13]. Similarly, detections of virus activity in sentinel chicken flocks in Victoria in 2008 were the first since 1974 [14]. An equine case of MVE also occurred in southeast Queensland in 2008 [10]. In 2011, following record levels of rainfall, widespread virus activity was once again observed across Australia, with MVEV detected in all mainland states, resulting in seventeen cases of human disease and numerous equine cases [11,15].

Four genotypes (G1-G4) of MVEV have been identified, based on limited genetic analyses using RNase oligonucleotide mapping [16] or gene sequencing of the E gene [17,18], 5’ terminal non-coding region [19], and NS5-3’non-coding region [20]. These studies showed that G1 is the predominant type on mainland Australia. The most recent isolates of MVEV from PNG also belong to G1 [18]. Genotype 2 consists of mosquito isolates from the northeast Kimberley region of northern Western Australia. G2 viruses have not been found outside this area, suggesting that this lineage occupies a unique and/or rarely sampled ecological niche [18,21], or may represent an incursion from the Indonesian archipelago [3,19]. Genotypes 3 and 4 comprise single strains of MVEV from PNG isolated from a human case in 1956 [8] and from mosquitoes in 1966 [22], respectively.

The single-stranded positive sense RNA genome of MVEV encodes three structural proteins (capsid, pre-membrane [prM] and envelope [Env]) and seven non-structural proteins (NS1, NS2A, NS2B, NS3, NS4A, NS4B and NS5) in the ORF. The prM and Env proteins have important biological roles in virus particle assembly and virus entry during infection of cells [23]. The Env protein is also the major target of neutralising antibodies during infection [24], and uncleaved prM, present in immature virus particles and released from infected cells, can elicit host immunity [25]. Both prM and Env genes exhibit relatively high levels of sequence variation, allowing robust phylogenetic resolution [26–29]. In the present study, we performed an expanded phylogenetic analysis of MVEV, including sixty-four new MVEV structural gene (prM-Env) sequences, in order to better understand the genetic relationships of earlier isolates with more recent circulating strains. In addition, to gain insights into the temporal and biological basis of MVEV evolution, molecular clock and selection analyses were undertaken.

## Materials and Methods

### Viruses

Virus strains were sourced from the culture collections of the authors. In addition, MVEV strains PNG6910, PNG6523 and CY1189 were kindly provided by Prof Roy Hall (University of Queensland). When required, one to two additional passages of virus stocks were performed in porcine stable equine kidney cells (Arbovirus Surveillance and Research Laboratory, University of Western Australia). MVEV strain 611W/WA/08 was identified in post-mortem clinical specimens taken from the lymph node, brain stem, cervical cord and cerebrum of a fatal human MVE case from Kununurra, Western Australia, in April 2008. Virus isolation from these specimens in cell culture was unsuccessful. Clinical specimens were kindly provided by Dr David Smith (PathWest Laboratory Medicine WA).

### RT-PCR and sequencing

Viral RNA was extracted from infected cell culture supernatant or clinical specimens using the QIAamp Viral RNA Mini kit or RNeasy Mini kit (QIAGEN). Reverse transcription of viral RNA was then performed using Superscript III (Invitrogen) and the oligonucleotide primer VD8, specific for the 3’untranslated region (3’UTR) of the virus genome [30]. Amplification of the complete prM and Env genes of MVEV was performed from viral cDNA using PCR SuperMix (Invitrogen) and overlapping oligonucleotide primers (S2 Table). Amplicons were directly sequenced at the Australian Genome Research Facility (Brisbane) using an AB3730xl capillary sequencer.

### Phylogenetic analyses

Viral sequences were aligned using ClustalW as implemented in MEGA6 [31]. Phylogenetic trees were constructed from either combined prM and Env genes (2004 nt) or individual prM (501 nt) or Env genes (1503 nt). Maximum Likelihood (ML) trees were estimated using PhyML v3.0 [32] using substitution models and rates among sites selected with JModelTest v2.1.5 [33]. Neighbour-Joining (NJ) trees were constructed using a maximum composite likelihood model with a gamma distribution using MEGA6. Reliability of the inferred trees was tested by the bootstrap method using 1000 replicates. All trees were rooted with analogous genes from JEV (GenBank accession no. AF217620), and visualised using FigTree v1.4.0. Pairwise distances were determined at the nucleotide and amino acid level for prM and/or Env gene sequences using the p-distance model in MEGA6.

In order to assess the temporal signal of the ML prM-Env phylogeny, which does not assume the operation of a molecular clock, a root-to-tip regression analysis was first performed using the Path-O-Gen (v1.3) program (http://tree.bio.ed.ac.uk/software/pathogen/). The Bayesian Markov Chain Monte Carlo (MCMC) method available in BEAST v1.8 [34] was then employed to estimate the evolutionary rates and divergence times from the most recent common ancestors (MRCA) using prM-Env sequences. BEAST analyses used a relaxed molecular clock (uncorrelated lognormal), the GTR nucleotide substitution model with no site heterogeneity and partitions at each codon position (1+2+3), and a Bayesian skyline coalescent tree prior [35]. MCMC chains were run for 50 million generations and convergence was assessed using Tracer software (v1.6; http://beast.bio.ed.ac.uk). Effective sample size values were greater than 200 for all parameters. A maximum clade credibility (MCC) tree across all sample trees generated by BEAST was computed using TreeAnnotator available in the BEAST package, with the first 10% of trees removed as burn-in. Posterior probability (PP) indicates the degree of support for each node on the tree, while statistical uncertainty in parameter estimates are reflected as the 95% highest probability density (HPD) values.

### Recombination analyses

Evidence of recombination between MVEV prM-Env genes was tested using the RDP3 program, which employs the RDP, Chimeara, BootScan, 3Seq, GENECONV, MaxChi, SiScan, and LARD methods to detect and characterise recombination events [36]. Sequences below a minimum genetic distance threshold of 0.006 (p-distance) were removed from the analyses since recombination between similar sequences is not detectable. Default settings were used for each method.

### Selection pressures within MVEV

Alignments of prM, Env or prM-Env genes for sixty-six MVEV strains were analysed using the DataMonkey server (http://www.datamonkey.org/), a webserver of the HyPhy package. The single likelihood ancestor counting (SLAC), fixed effects likelihood (FEL), internal FL (IFEL), and random effects likelihood (REL) codon-based maximum likelihood methods were employed to test for positive (diversifying) selection [37,38]. These methods calculate the nonsynonymous (amino-acid change)/synonymous (silent) ratio (dN/dS) for codons using NJ phylogeny and the general reversible nucleotide substitution (REV) model (Env and prM-Env) or TrN93 model (prM). In addition, the mixed effects model of evolution (MEME) [39] and fast unbiased Bayesian approximation (FUBAR) [40] methods were used to detect episodic and pervasive diversifying selection, respectively. Evidence of directional selection was also assessed using the directional evolution in protein sequences (DEPS) method [41] in HyPhy. The Jones, Taylor, Thornton (JTT) amino acid substitution model and NJ phylogeny were employed in the DEPS method.

### Homology modelling

Since the MVEV Env protein crystal structure has not yet been solved, we constructed a homology model to map amino acid residues and sequence motifs of interest. As there is a high degree of amino acid sequence identity between MVEV and JEV (~80%), the crystal structure of the JEV Env protein ectodomain was used as the template [PDB accession code 3P54; [42]]. This was performed using the Swissmodel structure homology server [43]. To visualise and identify selected residues and motifs on the resulting homology model, we used the UCSF Chimera package, v1.9 [44].

### Nucleotide sequence accession numbers

All sequences generated in this study were deposited in GenBank (Accession no. JN119755 to JN119814 for prM-Env gene sequences; S1 Table).

### Ethics statement

The animal work shown in S8 Table was conducted with the approval of the Curtin University Animal Ethics Committee (approval number: AEC_2011_64). All procedures were conducted according to the Australian code for the care and use of animals for scientific purposes [45]. Mice were euthanised by intraperitoneal injection of a lethal dose of pentobarbitol sodium (150 mg/kg body weight) followed by cervical dislocation.

## Results

### PrM and Env gene sequencing of selected MVEV strains

**Fig 1:**
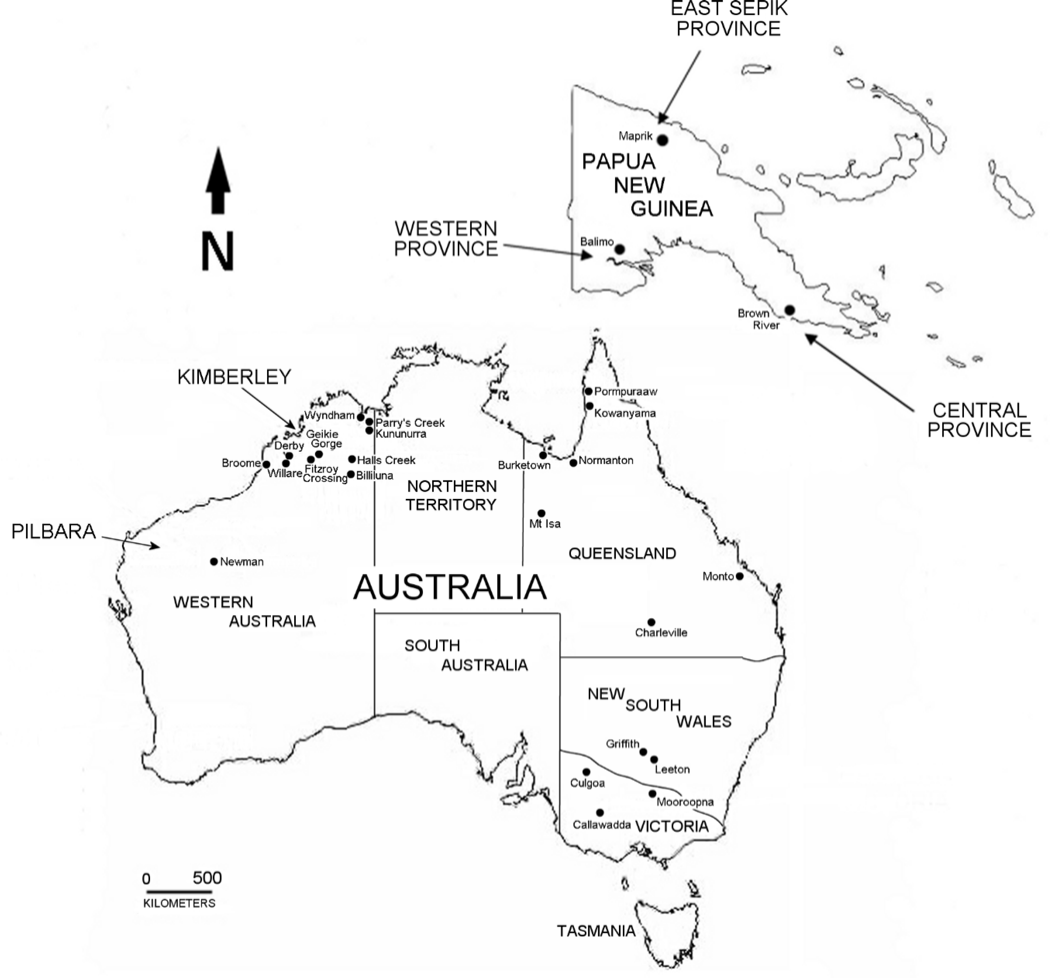
Map of Australia and Papua New Guinea showing the geographic origins of the MVEV strains used in this study. Australian states and territories are indicated, as well as selected regions or provinces.

Complete prM and Env genes encoded by sixty-six strains of MVEV were sequenced. Virus strains originated from the known geographic range of MVEV, encompassing north-western, eastern and south-eastern Australia, as well as PNG, and were isolated over a sixty year period from 1951 (S1 Table; Fig 1). Analogous gene sequences of the previously reported prototype strain MVE-1-51 [46] and the equine isolate V11-10 [47] were also included in this study. Pairwise comparison of partial or complete prM or Env gene sequences previously submitted to GenBank with sequences generated in this study revealed minor sequence inconsistencies for some strains; namely, NG156 (EF015076), 145663 (FJ037717), 145694 (FJ037721) and 145705 (FJ037722). In addition, the partial Env sequences for PNG6523 (EF015065) and PNG6910 (EF015066) matched with PNG6910 and PNG6523 sequences, respectively, from this study. In all cases, discrepancies were confirmed by re-sequencing from the earliest passage of virus culture available.

### Phylogenetic analysis of MVEV prM and Env genes

**Fig 2:**
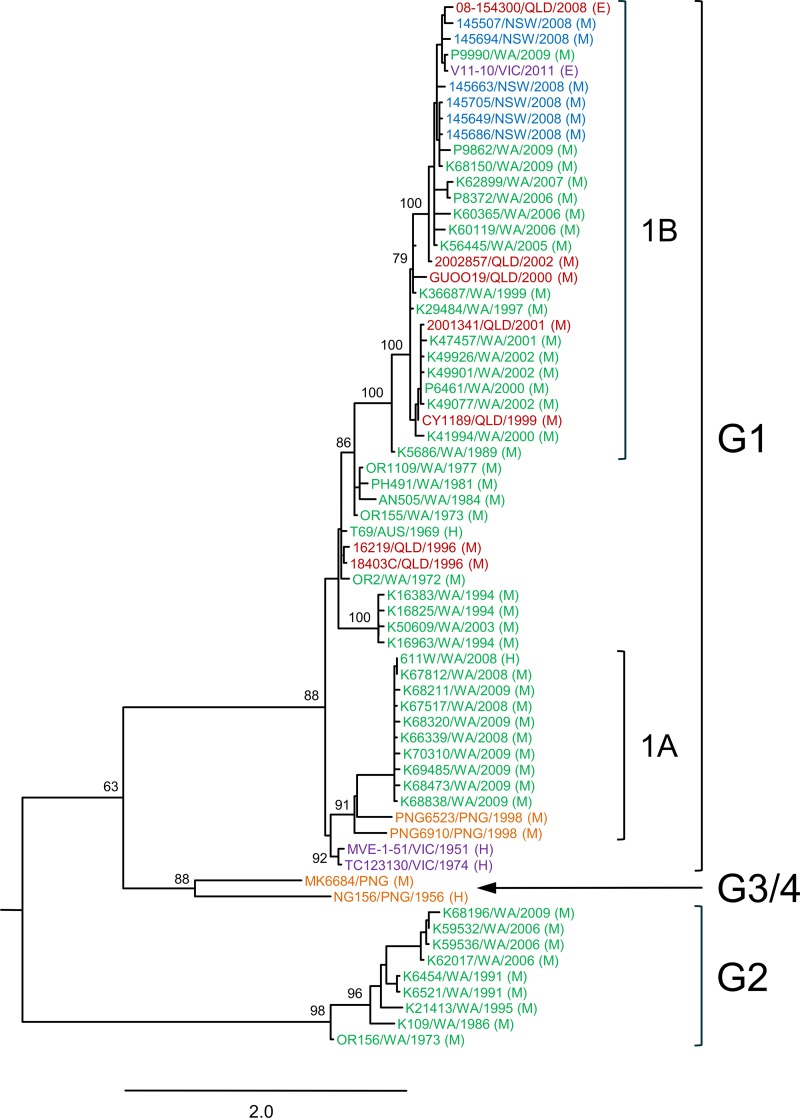
ML phylogenetic tree estimated using MVEV prM-Env gene sequences. Genotypes and sub-lineages are shown and geographic origin of strains is indicated by font colour: New South Wales, blue; Queensland, red; Victoria, purple; Western Australia, green; Papua New Guinea, orange. The tree was estimated using a general time-reversible model of nucleotide substitution with a gamma distribution and invariant sites. Numbers at the nodes represent bootstrap support as a percentage of 1000 replicates; only values ≥50% are shown. The scale bar indicates 2 nucleotide substitutions per site. The tree was rooted with the prM-Env sequence of JEV, however this has been removed to improve visual resolution of the tree.

In phylogenetic analyses, each of the four previously identified genotypes could be readily identified using the prM and Env genes, either individually or in combination. The exception was G2, which was not well resolved using the prM gene (S1 Fig). The different phylogenetic methods employed estimated similar trees. However, the NJ and ML methods differed with respect to the branching positions of the G2, G3/G4 lineages in the Env and prM-Env trees (S1 Fig). Bootstrap support at the corresponding nodes was also poor (<70%), such that the relative branching positions of G2 and G3/G4 could not be reliably inferred. The phylogenetic tree estimated using the ML method and combined prM-Env sequences is shown in Fig 2, as representative of this analysis. G2 occupies the most divergent lineage and is made up of isolates from mosquitoes trapped in the Kimberley region of Western Australia between 1973 and 2009. Genotypes 3 and 4 form a well-supported clade, internal to G1 and G2 in the ML tree; no new members of G3 or G4 were found. G1 is the largest group, comprising 54 strains (82% of the sample set), indicating its dominance over the sampling period.

Pairwise distances within and between genotypes calculated using the prM and Env genes reflect the inferred phylogenies (S3 Table). Genotype 1 strains have high levels of genetic identity to each other within the prM (≥96.2% nt) and Env genes (≥93.7% nt). Strains within G2 have slightly higher levels of nt identity (prM: ≥97.8%; Env: ≥96.2%), but are the most divergent when compared to other genotypes (prM: ≥86.8%; Env: ≥84.8%). As for previous studies [16,18], G3 and G4 are the most closely-related of the genotypes (prM: 94% nt, 99.4% aa; Env: 93% nt, 99.2% aa), and have comparable levels of identity to G1 and G2. Genotypes could be defined by nt p-distance (%) cut-off values of 6.0% (prM), 7.0% (Env), and 6.7% (prM-Env).

### Recent circulating strains of MVEV belong to distinct sub-lineages of G1

Phylogenetic analyses revealed two distinct sub-lineages within G1, designated G1A and G1B, based on their relative age of divergence (see Evolutionary history, below). These sub-lineages comprise the most recent strains of MVEV. G1A includes Western Australian strains from mosquitoes collected in 2008 and 2009 and one human case of MVE from 2008, as well as the most recent strains from PNG, isolated in 1998. G1A shares common ancestry with early isolates from Victoria (MVE-1-51 and TC123130). As for G2 viruses, all recent Australian strains in the G1A sub-lineage were isolated from the Kimberley region. In contrast, G1B is the most geographically diverse genotype and comprises strains isolated from Western Australian, Queensland, New South Wales and Victoria between 1989 and 2011. Both G1A and G1B sub-lineages are genetically homogenous (S3 Table): G1A has ≥98.0% nt (≥99.4% aa) identity between strains for the prM gene and ≥97.3% nt (≥99.6% aa) identity for Env; G1B strains share ≥98.8% nt (≥98.2% aa) identity for prM and ≥97.6% nt (≥99.0% aa) for Env.

Several intermediate sub-lineages within MVEV G1 were also observed, comprising Queensland isolates from 1996 and early isolates (1969–84) from Western Australia (Figs 2 and S1). Of these, only two could be reliably resolved: the first comprised four strains from the Kimberley region of Western Australia, isolated between 1994 and 2003 (K16383, K16825, K16963 and K50609), the other was made-up of three strains from the Kimberley and Pilbara regions, isolated between 1977 and 1984 (AN505, OR1109 and PH491).

### Sequence analysis of the prM and Env proteins: Genotype-defining amino acid differences and conservation in biologically important motifs

**Fig 3:**
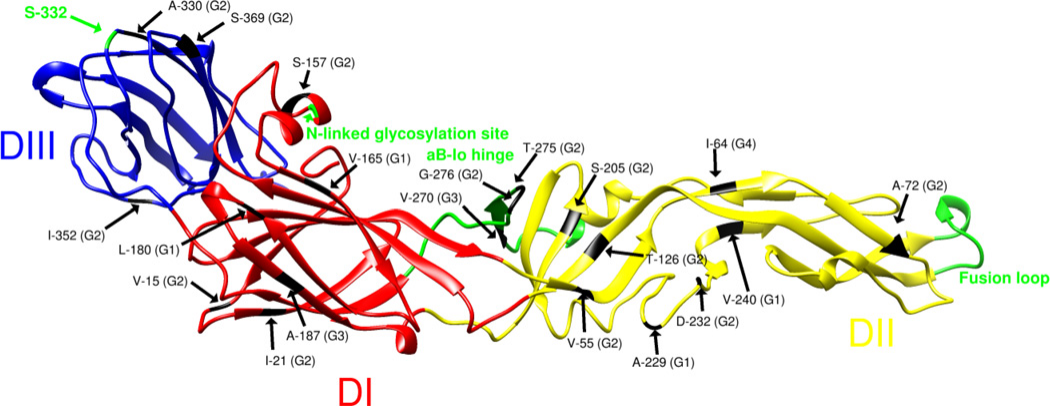
Location of genotype-defining amino acid residues and motifs in the ectodomain of the MVEV Env protein, based on the crystal structure of JEV [42]. The three domains of the Env protein are shown in red (DI), yellow (DII) and blue (DIII). The fusion loop is coloured green, and the N-linked glycosylation site at position 154, the hypervariable domain in DII and the hinge connecting the αB helix of DII and the I_o_ β-strand of DI are indicated.

For each genotype, one or more amino acid residues were found to be unique to, and defining of, all virus strains belonging to that genotype (S4 Table). Using the Env protein crystal structure of the closely-related JEV [42], the locations of these amino acids were modeled (Fig 3). The flavivirus Env protein is comprised of three structural domains: a central β-barrel (DI), an elongated dimerisation region (DII) and a C-terminal immunoglobulin-like domain (DIII) [48]. The soluble ectodomain of the Env protein is tethered to the viral membrane by a transmembrane region.

For G1, V165 (DI), A229 (DII) and V240 (DII) of the Env protein are unique to this genotype. Sub-lineages 1A and 1B can be further differentiated by single conservative amino acids substitutions in DI of Env (L180) or the prM (V24), respectively. Fifteen amino acids located in each of the structural domains and the transmembrane region of the Env protein of G2 viruses are defining of this genotype (S4 Table). Genotype 3 is defined by four amino acids encoded in the prM (H77) and Env protein in domains I and II and the stem (A187, V270 and I442), while G4 encodes only a single unique amino acid (I64) in DII. Two regions within the Env protein also encode sequences that are differentiating of the genotypes (S2 Fig). The region encompassing positions 228 to 232 in DII corresponds to a hypervariable domain previously identified for flaviviruses [49]. Within this region, G1 encodes the sequence motif PAST/SE, G2 encodes PSN/STD, and G3 and G4 encode PSSTE (S2A Fig).

Other regions and motifs of the prM and Env proteins known to be biologically important or to encode virulence determinants of MVEV or other flaviviruses were conserved within this species. In the prM protein, these included the flavivirus conserved region at position 61 to 69 [50] and tyrosine 78 [51], both involved in virus assembly. The canonical furin cleavage site [52], and the helix domain (prM 114–130), which interacts with the Env protein in immature particles [53], were also conserved. In the Env protein, the glycosylation site (NYS) at positions 154–157 [54,55], the receptor binding site in the F-G loop of DIII [positions 387–392; [48,56]], and the fusion peptide in DII [57] were all conserved among the MVEV strains sequenced. Histidine residues proposed to be involved in the structural transition leading to membrane fusion [58] were also conserved.

### Positive selection at position S332G of the Env protein

Selection analyses were performed to assess whether the genotype-defining amino acids identified above were selected during the evolution of MVEV. The dN/dS ratio of nucleotide substitutions was estimated to be 0.052 in the DataMonkey analysis, indicating predominantly negative (purifying) selection (S5 Table), consistent with findings for other flaviviruses [59–62]. A single (non-conservative) amino acid substitution in the Env protein (S332G) was found to be under positive (diversifying) selection in five of the six methods employed. This position is located in the B-C loop of DIII and is encoded by fourteen strains of G1 viruses isolated from pools of *Cx*. *annulirostris* and *Ae*. *normanensis* in Western Australia and Queensland between 1977 and 2009 (S6 Table). The S332G substitution is found in strains belonging to the recently emerged G1A and G1B, as well as intermediate lineages within G1, and does not therefore correlate with phylogenetic sub-lineage. One site in the prM protein and seventeen sites in the Env protein were found to be under directional evolution (S7 Table), including S332G; thirteen of these were identified as genotype-defining.

### No evidence for recombination in the MVEV prM-Env genes

Evidence for homologous recombination, with breakpoints in the Env gene, has been reported for JEV and other flaviviruses, such as dengue virus (DENV) and Saint Louis encephalitis virus, based on database-derived sequences [63,64] or from cloned cDNA from clinical and mosquito isolates [65,66]. Experimental evidence of recombination between JEV genomes has also been reported [67,68]. In the present study, no evidence of recombination was detected in MVEV prM-Env genes using eight different computational methods implemented in the RDP3 program [36]. This is consistent with the finding of no major incongruence in phylogenetic groupings when trees generated from prM or Env genes were compared (S1 Fig).

### Evolutionary history of MVEV

**Fig 4:**
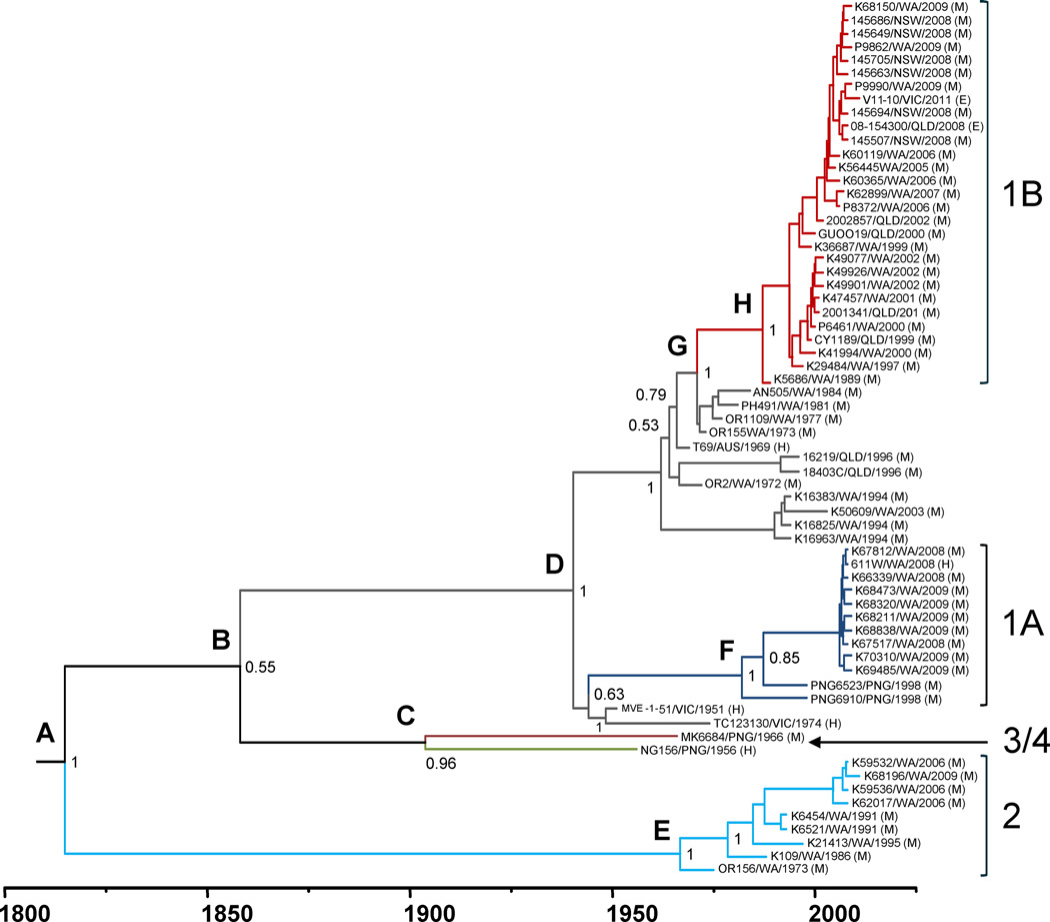
Bayesian MCC phylogenetic tree for MVEV prM-Env sequences. Divergence times and branch lengths are drawn according to the time scale bar (in years), such that the tips of the tree correspond to sampling date of each virus strain. Branches and ancestral lineage of each genotype and sub-lineage are coloured blue (G1A), red (G1B), grey (remaining G1 strains), light blue (G2), green (G3) and dark red (G4). Posterior probability values are shown at the nodes. Letters at the upper left of each node correspond to divergence times and substitution rates shown in Table 1.

The root-to-tip regression analysis of the MVEV prM-Env sequences demonstrated a high level of clock-like behavior in the tree (*R* ^*2*^ = 0.65) and therefore justified a more in-depth analysis of lineage evolution. To do this, a Bayesian MCMC analysis was performed to estimate time to MRCA and evolutionary rates for MVEV genotypes. The MCC tree for the prM-Env sequences is shown in Fig 4. The MRCA for all genotypes of MVEV was estimated to circulate between 1703 and 1902 (mean: 1814; Table 1). G2 emerged directly from this ancestral virus, with further evolution of recent isolates occurring between 1953 and 1973 (mean: 1965). The ancestral lineage of genotypes 1, 3 and 4 evolved between 1794 and 1914 (mean: 1858), and G3 and G4 later diverged into distinct lineages between 1860 and 1944 (mean: 1904). The MRCA of G1 evolved between 1930 and 1949 (mean: 1940), just prior to the first isolation of MVEV, belonging to this genotype, in the 1950–51 epidemic in southeast Australia [69]. The sub-lineages of G1A and G1B were the most recent to emerge: G1A between 1969 and 1994 (mean: 1982) and G1B between 1984 and 1989 (mean: 1987).

**Table 1:** Divergence times and evolutionary rates for genotypes and sub-lineages of MVEV estimated by Bayesian MCMC analysis.

Node^a^	Genotype	Divergence date		Mean substitution rate (substitutions/site/year)	
		Year	95% HPD range		95% HPD range
A	All	1814	1703–1902	6.04 × 10^−4^	4.02 × 10^−4^ to 8.03 × 10^−4^
B	G1+3+4	1858	1794–1914	6.35 × 10^−4^	1.50 × 10^−3^ to 9.72 × 10^−5^
C	G3+4	1904	1860–1944	5.68 × 10^−4^	1.27 × 10^−3^ to 8.67 × 10^−5^
D	G1	1940	1930–1949	8.40 × 10^−4^	1.68 × 10^−3^ to 9.72 × 10^−5^
E	G2	1965	1953–1973	9.60 × 10^−4^	1.97 × 10^−3^ to 9.28 × 10^−5^
F	G1A	1982	1969–1994	2.39 × 10^−4^	4.01 × 10^−4^ to 9.95 × 10^−5^
G	MRCA to G1B	1971	1968–1973	7.92 × 10^−4^	1.55 × 10^−3^ to 2.35 × 10^−4^
H	G1B	1987	1984–1989	8.04 × 10^−4^	1.19 × 10^−3^ to 4.60 × 10^−4^

^a^Nodes are as indicated in the MCMC tree shown in Fig 3.

Nucleotide substitution rates were also estimated from the Bayesian MCMC analysis. The mean rate of nucleotide substitutions within the prM-Env genes for all MVEV strains was 6.04 × 10^−4^ substitutions per site per year (95% HPD: 4.02 × 10^−4^ to 8.03 × 10^−4^). A low level of heterogeneity in evolutionary rates, as estimated by the MCMC analysis, was observed between individual genotypes and ancestral lineages (Table 1). Although rates varied from 2.39 × 10^−4^ (G1A) to 9.60 × 10^−4^ (G2) substitutions per site per year, uncertainty intervals (95% HPD) for all lineages were overlapping. These rates are comparable to those estimated for other flaviviruses belonging to the JEV serogroup [60,70–72].

Regression analysis of the prM-Env gene phylogeny supported the above results from the MCMC analysis. The estimated time to the MRCA was the year 1800 (*R* ^*2*^ = 0.65) for all strains and the substitution rate was 6.68 × 10^−4^ nucleotide substitutions per site per year.

## Discussion

In this study, we aimed to expand previous phylogenetic analyses of MVEV using the two major structural genes and the largest sequence data set compiled to date. We also investigated the evolutionary history of MVEV using selection and molecular clock analyses. We confirmed previous findings that G1 is the dominant genotype of this species and identified two distinct sub-lineages (designated G1A and G1B) that have recently emerged. G2 was shown to be the oldest lineage, while no additional members of G3 and G4 were found. This study clearly demonstrated that all contemporary lineages of MVEV have been isolated from Kimberley region of Western Australia, which supports the previous contention that the enzootic focus for this virus resides in northwestern Australia, at least since the early 1970s following construction of the Ord River irrigation area [3,73].

Of the two G1 sub-lineages of MVEV identified in this study, G1B is the most widely transmitted and, for Australian strains, the only contemporary genotype found outside northwestern Australia; viruses belonging to this group have been isolated from the eastern Australian states. G1A comprised both Australian and PNG strains, the latter forming separate lineages within this group (Fig 2). Phylogenetic analyses showed that these viruses diverged from a common ancestral strain, further supporting evidence for virus movement between northwestern Australia and southern PNG [18]. Despite this, and in contrast to G1B, the Australian G1A viruses have only been isolated from the Kimberley region of WA. A similar geographic restriction was observed for G2 viruses, and it has been suggested that this lineage occupies an ecological niche that underlies its range [18,21]. Australian G1A viruses may also be subjected to ecological or biological restrictions. Similarly, G3 or G4 viruses appear to be either confined to the New Guinea Island or have become extinct. No new members of these types were identified in this study. However, in this regard, it is notable that surveillance activities for MVEV and other flaviviruses in PNG are rarely undertaken, and due to the paucity of isolates from this country, the circulation of additional genotypes cannot be discounted.

The observation of dominant and minority circulating genotypes for MVEV has also been found for other members of the JEV group. Notably, GIII of JEV was dominant throughout northern and western Asia for several decades until it was displaced in the early 1990s by GI, which is now the major circulating lineage [74–78]. In contrast, JEV GIV viruses have only been isolated from mosquitoes and appear to be geographically confined to Indonesia [78], analogous to the apparent restricted circulation of MVEV G2 in the Kimberley region of Western Australia. Thus, these minority lineages, which have no known disease association in either humans or animals, may represent low pathogenic types. However, given their close relationships with pathogenic viruses, and the propensity of flaviviruses to rapidly evolve and adapt to new environments and hosts, they may represent potential pathogens.

One or more unique amino acids encoded in the prM and Env proteins were found to define each MVEV genotype (S4 Table). Given the importance of these proteins in the virus replication cycle and host immune response, these differences may underlie the observed variation in geographic distribution of genotypes and sub-lineages. Of potential significance for G1 viruses is Env 229, which is located on the ‘h’ loop of DII within the flavivirus hypervariable domain [49], a region containing both type- and group reactive flavivirus epitopes [79,80]. Thus, A229 may have been selected as an escape from neutralization mutation that has conferred a fitness advantage to G1 MVEV. Since A229 is conserved within both G1A and G1B sub-lineages, the factors determining the observed differences in their transmission patterns in Australian may lie in other regions of the genome. Full genome sequencing of G1A strains may reveal biologically significant differences to the genome of G1B MVEV [47]. It may be speculated that G1B viruses have a replicative advantage enabling more rapid and widespread transmission. Viruses of this genotype may be more efficiently transmitted by vector mosquitoes. This is believed to be a contributing factor to the emergence of the dominant WNV WN02 genotype in North America between 2001 and 2004, which was found to be transmitted earlier and more efficiently than the pre-existing NY99 genotype virus [81].

For G2 viruses, several of the observed unique amino acids have potential biological significance. Residues at positions 72, 126, 275, 276, 330 and 369 are predicted to be exposed on the virus surface (Fig 3) [42,48] and have been associated with biological function and antigenicity. Env 126 has been shown experimentally to be a determinant of neurovirulence in mice for DENV2 [82]; and residues 330 and 369 are located in loops of DIII involved in receptor attachment [48,83]. Moreover, amino acids at positions 275 and 276 are located in one of four putative hinges connecting DI and DII that are thought to play a critical structural role during pH-dependent membrane fusion [42,48,84]. Mutations at the adjacent position 277 have also been shown to reduce MVEV haemagglutination and attenuate neuroinvasiveness in mice [85,86]. It has been previously proposed that the unique amino acids encoded by G2 viruses may affect their replicative fitness and virulence, thereby restricting their transmission via vector mosquitoes and/or water birds [18]. Our findings support this hypothesis, as do *in vivo* studies using the mouse model of neurotropism: we and others have shown that G2 viruses, including early (OR156) and recent (K59532) strains, have a low virulence phenotype following intraperitoneal inoculation ([87]; S8 Table).

The unique amino acid residues encoded by G2 virus strains at positions 126, 275, 276 and 330 make-up neutralizing epitopes of MVEV (126, 276), WNV (330) and JEV (126, 275) [80,88–91]. We predict these residues may also distinguish G2 viruses antigenically from viruses belonging to other genotypes. Similarly, position 332 in the Env protein, found to be under positive selection for some G1 strains (S6 Table), is found on the distal lateral surface of DIII, which protrudes from the virion surface and is a known hot-spot for potent flavivirus neutralising epitopes [90,92–95]. Mutations at position 332 of WNV and JEV (MVEV numbering) can reduce or abolish antibody binding [90–92]. MVEV strains encoding the S332G substitution may have escaped from antibody neutralisation and are antigenically different at this site compared to other MVEV strains.

The MCMC analysis suggests that MVEV evolved from its MRCA approximately 200 years ago, at around 1814 (Table 1; Fig 4). In relation to other JEV group viruses circulating in Southeast Asia, this MRCA date is slightly earlier than that of the Australian subtype of WNV, Kunjin virus (WNV-KUNV), which was estimated to be approximately 1851 [71], but much later than JEV, estimated to be around the mid-1500s [72,78]. Since its emergence in the Australasian region, WNV-KUNV therefore appears to have co-evolved with MVEV, in contrast to JEV, which is believed to have emerged in this region only 20–30 years ago [61,96]. The earliest recorded outbreaks of encephalitis in eastern Australia occurred between 1917 and 1925, and are believed to have been caused by MVEV, based on clinical and epidemiological evidence [97]. From the MCMC analysis in this study, these outbreaks appear to have been caused by a virus belonging to the ancestral lineage of G1 (Fig 4), which diverged at around 1940 prior to the MVE epidemic in the early 1950s. Virus isolates from this epidemic and the 1974 epidemic belong to an ancestral lineage of G1A, which was then displaced by intermediate lineages and the progenitor of G1B. The latter subtype emerged at some time between 1968 and 1973. This is significant since this coincides with construction of Stage 2 of the Ord River Irrigation scheme in the northeast Kimberley. Stage 2 involved building the Ord River dam to create Lake Argyle, a major water reservoir, and was completed in 1972. This resulted in profound changes to the local ecosystem, and it has been hypothesised that this led to MVEV becoming established in enzootic cycles in this region [73]. Our findings suggest that G1B subsequently became the dominant and most widespread lineage of MVEV in Australia. We propose that the changes to the ecosystem in the northeast Kimberley facilitated the emergence of this lineage, via increased populations of waterbirds and *Culex* species mosquitoes, and the creation of year-round conditions suitable for maintaining virus transmission cycles.

Currently, only four full length genomes of MVEV have been published [86,98]. Additional full length genomes are expected to allow more accurate and comprehensive evolutionary analyses of MVEV. Further sequencing of earlier isolates may also provide a greater level of confidence to the MCMC analysis, particularly for older divergence points. Continued surveillance will also be necessary to track the ongoing evolution of this virus. It is expected that sub-lineages G1A and G1B will continue to diverge genetically and potentially antigenically, with possible consequences to the sensitivity of existing laboratory diagnostics. Of interest will be the relative pattern of spread of each of these lineages. Are G1A and G2 viruses found outside northwestern Australia? And if not, what are the biological and ecological drivers of this phenomenon? The isolation and characterisation of MVEV strains from regions north of Australia, such as PNG and Indonesia, will also shed light on the diversity of MVEV circulation and patterns of spread to mainland Australia.

## Supporting Information

S1 Table Details of MVEV strains, detected or isolated in Australia and PNG between 1951 and 2011, used for genetic analyses in this study. (DOCX)

S2 Table Oligonucleotides employed for amplification of pre-membrane and envelope genes of Murray Valley encephalitis virus. (DOCX)

S3 Table Pairwise distances between genotypes and subgenotypes of Murray Valley encephalitis virus for the pre-membrane (prM) and envelope genes. (DOCX)

S4 Table Unique amino acids in the pre-membrane and envelope protein sequences of MVEV that define genotype or sub-lineage. (DOCX)

S5 Table Evidence for positive and negative selection in the prM and Env genes (668 amino acids) of Murray Valley encephalitis using six different methods implemented in the DataMonkey server of the HyPhy software package. (DOCX)

S6 Table Murray Valley encephalitis virus strains encoding the S332G amino acid substitution in the envelope protein. (DOCX)

S7 Table Amino acid sites within the prM and Env proteins under directional selection. Amino acids corresponding to genotype-defining residues are indicated by (*).
(DOCX)

S8 Table Virulence of Murray Valley encephalitis virus strains, representing genotypes 1 and 2, in 18-day old Swiss mice following intraperitoneal inoculation. (DOCX)

S1 Fig Phylogenetic analysis of MVEV using prM (A, B), Env (C, D) and prM-Env (E, F) gene sequences. NJ trees (A, C and E) were estimated with the maximum composite likelihood model with a gamma distribution. ML trees (B, D and F) were estimated using a general time-reversible model of nucleotide substitution with a gamma distribution and invariant sites. Numbers at the nodes represent bootstrap support as a percentage of 1000 replicates; only values ≥50% are shown. The scale bar indicates nucleotide substitutions per site. Each tree was rooted with the analogous sequence of JEV, however this has been removed to improve visual resolution of the tree.
(PDF)

S2 Fig The hypervariable region (A) and DI-DII hinge motif (B) of the Envelope protein encoded by representative strains of Murray Valley encephalitis virus. Identical amino acids are colour-shaded. The symbols below the alignments indicate identical amino acids (*), strongly conserved (:), and weakly conserved (.) amino acids.
(PDF)
