# Supplementary material for: The Molecular Epidemiology and Evolution of Murray Valley Encephalitis Virus: Recent Emergence of Distinct Sub-lineages of the Dominant Genotype 1
Source: PLoS Negl Trop Dis. 2015 Nov 24;9(11):e0004240. doi: 10.1371/journal.pntd.0004240 (PMC4657991; doi:10.1371/journal.pntd.0004240)
Supplement: S1 Table — (DOCX) [file pntd.0004240.s001.docx]

**S1 Table. Details of MVEV strains, detected or isolated in Australia and PNG between 1951 and 2011, used for genetic analyses in this study.**

| **Strain** | **Year of isolation** | **Location^a^** | **Species of Origin** | **GenBank accession number** |
| --- | --- | --- | --- | --- |
| MVE-1-51 | 1951 | Mooroopna, Victoria | Human brain | NC000943 |
| NG156 | 1956 | Brown River, Central Province, PNG | Human brain | JN119801 |
| MK6884 | 1966 | Maprik, East Sepik Province, PNG | Mixed Culicines | JN119800 |
| T69 | 1969 | Northern Australia | Human brain | JN119813 |
| OR2 | 1972 | Kununurra, NE Kimberley, WA | *Culex annulirostris* | JN119805 |
| OR155 | 1973 | Kununurra, NE Kimberley, WA | *Cx. annulirostris* | JN119803 |
| OR156 | 1973 | Kununurra, NE Kimberley, WA | *Cx. annulirostris* | JN119804 |
| TC123130 | 1974 | Culgoa, NW Victoria | Human brain | JN119814 |
| OR1109 | 1977 | Kununurra, NE Kimberley, WA | *Cx. annulirostris* | JN119802 |
| PH491 | 1981 | Newman, E Pilbara, WA | *Cx. annulirostris* | JN119810 |
| AN505 | 1984 | Kununurra, NE Kimberley, WA | *Aedes normanensis* | JN119768 |
| K109 | 1986 | Kununurra, NE Kimberley, WA | *Cx. annulirostris* | JN119770 |
| K5686 | 1989 | Kununurra, NE Kimberley, WA | *Cx. annulirostris* | JN119784 |
| K6454 | 1991 | Kununurra, NE Kimberley, WA | *Cx. annulirostris* | JN119788 |
| K6521 | 1991 | Kununurra, NE Kimberley, WA | *Anopheles bancroftii* | JN119789 |
| K16825 | 1994 | Billiluna, SE Kimberley, WA | *Cx. annulirostris* | JN119772 |
| K16383 | 1994 | Wyndham, NE Kimberley, WA | *Cx. annulirostris* | JN119771 |
| K16963 | 1994 | Billiluna, SE Kimberley, WA | *Cx. annulirostris* | JN119773 |
| K21413 | 1995 | Kununurra, NE Kimberley, WA | *Cx. pullus* | JN119774 |
| 16219 | 1996 | Charleville, SW Queensland | *Cx. annulirostris* | JN119761 |
| 18403C | 1996 | Mitchell River, West Cape York Peninsula, Queensland | *Cx. annulirostris* | JN119762 |
| K29484 | 1997 | Kununurra, NE Kimberley, WA | *Cx. pullus* | JN119775 |
| PNG6523 | 1998 | Balimo, Western Province, PNG | *Cx. sitiens* group | JN119811 |
| PNG6910 | 1998 | Balimo, Western Province, PNG | *Cx. sitiens* group | JN119812 |
| CY1189 | 1999 | Pormuraaw, West Cape York Peninsula, Queensland | *Cx. sitiens* group | JN119769 |
| K36687 | 1999 | Kununurra, NE Kimberley, WA | *Cx. annulirostris* | JN119776 |
| K41994 | 2000 | Billiluna, SE Kimberley, WA | *Cx. annulirostris* | JN119777 |
| P6461 | 2000 | Newman, E Pilbara, WA | *Cx. annulirostris* | JN119806 |
| GU0091 | 2000 | Normanton, NW Queensland | *Cx. annulirostris* | JN119765 |
| 2001341 | 2001 | Mt Isa, NW Queensland | *Cx. annulirostris* | JN119763 |
| K47457 | 2001 | Derby, NW Kimberley, WA | *Cx. annulirostris* | JN119778 |
| K49077 | 2002 | Billiluna, SE Kimberley, WA | *Cx. annulirostris* | JN119779 |
| 2002857 | 2002 | Burketown, NW Queensland | *Cx. annulirostris* | JN119764 |
| K49926 | 2002 | Broome, W Kimberley, WA | *Cx. annulirostris* | JN119781 |
| K49901 | 2002 | Broome, W Kimberley, WA | *Cx. annulirostris* | JN119780 |
| K50609 | 2003 | Billiluna, SE Kimberley, WA | *Ae. normanensis* | JN119782 |
| K56445 | 2005 | Parry's Creek, NE Kimberley, WA | *Cx. annulirostris* | JN119783 |
| K59532 | 2006 | Fitzroy Crossing, W Kimberley, WA | *Cx. annulirostris* | KC206092 |
| K59536 | 2006 | Fitzroy Crossing, W Kimberley, WA | *Cx. pullus* | KC206093 |
| K60119 | 2006 | Halls Creek, SE Kimberley, WA | *Aedes* (*Macleaya*) species | JN119785 |
| K62017 | 2006 | Broome, W Kimberley, WA | *Cx. annulirostris* | KC206094 |
| K60365 | 2006 | Kununurra, NE Kimberley, WA | *Cx. annulirostris* | JN119786 |
| P8372 | 2006 | Newman, E Pilbara, WA | *Cx. annulirostris* | JN119807 |
| K62899 | 2007 | Kununurra, NE Kimberley, WA | *Cx. annulirostris* | JN119787 |
| 611W/WA/08 | 2008 | Kununurra, NE Kimberley, WA | Human brain | KM259934 |
| 08-154300 | 2008 | Monto, SE Queensland | Horse brain | JN119766 |
| 145507 | 2008 | Griffith, SW New South Wales | *Cx. annulirostris* | JN119759 |
| 145649 | 2008 | Griffith, SW New South Wales | *Cx. annulirostris* | JN119758 |
| 145663 | 2008 | Griffith, SW New South Wales | *Cx. annulirostris* | JN119757 |
| 145686 | 2008 | Leeton, SW New South Wales | *Cx. annulirostris* | JN119756 |
| 145694 | 2008 | Leeton, SW New South Wales | *Cx. annulirostris* | JN119755 |
| 145705 | 2008 | Leeton, SW New South Wales | *Cx. annulirostris* | JN119760 |
| K66339 | 2008 | Kununurra, NE Kimberley, WA | *Cx. annulirostris* | JN119790 |
| K67517 | 2008 | Willare, NW Kimberley, WA | *Cx. annulirostris* | JN119791 |
| K67812 | 2008 | Broome, W Kimberley, WA | *Cx. annulirostris* | JN119792 |
| K68150 | 2009 | Fitzroy Crossing,  W Kimberley, WA | *Cx. annulirostris* | JN119793 |
| K68196 | 2009 | Fitzroy Crossing,  W Kimberley, WA | *Cx. annulirostris* | KC206095 |
| K68211 | 2009 | Geikie Gorge, W Kimberley, WA | *Ae. (Macleaya)* species | JN119794 |
| K68320 | 2009 | Billiluna, SE Kimberley, WA | *Cx. annulirostris* | JN119795 |
| K68473 | 2009 | Wyndham, NE Kimberley, WA | *Cx. annulirostris* | JN119796 |
| K68838 | 2009 | Kununurra, NE Kimberley, WA | *Cx. annulirostris* | JN119797 |
| K69485 | 2009 | Kununurra, NE Kimberley, WA | *Cx. pullus* | JN119798 |
| K70310 | 2009 | Broome, W Kimberley, WA | *Cx. annulirostris* | JN119799 |
| P9862 | 2009 | Newman, E Pilbara, WA | *Cx. annulirostris* | JN119808 |
| P9990 | 2009 | Newman, E Pilbara, WA | *Cx. annulirostris* | JN119809 |
| V11-10 | 2011 | Callawadda, VIC | Horse brain | JX123032 |

^a^NSW, New South Wales; PNG, Papua New Guinea; QLD, Queensland; WA, Western Australia
