# Supplementary material for: The Molecular Epidemiology and Evolution of Murray Valley Encephalitis Virus: Recent Emergence of Distinct Sub-lineages of the Dominant Genotype 1
Source: PLoS Negl Trop Dis. 2015 Nov 24;9(11):e0004240. doi: 10.1371/journal.pntd.0004240 (PMC4657991; doi:10.1371/journal.pntd.0004240)
Supplement: S2 Table — (DOCX) [file pntd.0004240.s002.docx]

**S2 Table. Oligonucleotides employed for amplification of pre-membrane and envelope genes of Murray Valley encephalitis virus.**

| Primer name | Sequence (5’🡪3’) | Position in genome^a^ |
| --- | --- | --- |
| MVE-S1  MVE-A1  MVE-S2  MVE-A2  MVE-S3  MVE-A3 | GATTGATGTGGTGAACAAAAGGGG  GTTTRTCAGCRGCCATGATGG  GYAGCCGTGAYTTYATTGAAGG  GHRAACTCGACTGGTAYGGCTCCAG  GGAGTTTGAAGAGCCACATG  TRAGCTCYCTYCTGGTGATG | 368-391  1086-1066  991-1012  1795-1771  1691-1710  2514-2495 |

^a^Based on Genbank accession number NC_000943.
