# Supplementary material for: The Molecular Epidemiology and Evolution of Murray Valley Encephalitis Virus: Recent Emergence of Distinct Sub-lineages of the Dominant Genotype 1
Source: PLoS Negl Trop Dis. 2015 Nov 24;9(11):e0004240. doi: 10.1371/journal.pntd.0004240 (PMC4657991; doi:10.1371/journal.pntd.0004240)
Supplement: S3 Table — (DOCX) [file pntd.0004240.s003.docx]

**S3 Table. Pairwise distances between genotypes and subgenotypes of Murray Valley encephalitis virus for the pre-membrane (prM) and envelope genes.**

| **Genotype/ subgenotype** | **% range of nucleotide (amino acid) identities** | |
| --- | --- | --- |
|  | **prM** | **Envelope** |
| Within G1 | 96.2 (97.6) - 100 | 93.7 (98.4) - 100 |
| Within G1A | 98.0 (99.4) - 100 | 97.3 (99.6) - 99.9 (100) |
| Within G1B | 98.8 (98.2) - 100 | 97.6 (99.0) - 99.9 (100) |
| Between G1A & G1B | 95.4 (97.6) - 96.4 (99.4) | 93.7 (98.6) - 95.3 (99.4) |
| Between G1 & G2 | 86.8 (97.6) - 89.4 (100) | 84.8 (94.6) - 87.1 (95.6) |
| Between G1 & G3 | 89.6 (97.6) - 91.8 (99.4) | 88.3 (97.2) - 89.9 (98.2) |
| Between G1 & G4 | 89.0 (98.2) - 91.0 (100) | 88.3 (97.6) - 90.6 (98.6) |
| Within G2 | 97.8 (99.4) - 100 | 96.2 (98.6) - 99.8 (100) |
| Between G2 & G3 | 87.0 (98.8) - 88.0 (99.4) | 87.0 (95.0) - 86.3 (95.2) |
| Between G2 & G4 | 86.8 (99.4) - 88.0 (100) | 86.8 (95.4) - 87.1 (95.6) |
| Between G3 & G4 | 94.0 (99.4) | 93.0 (99.2) |
