# Supplementary material for: The Molecular Epidemiology and Evolution of Murray Valley Encephalitis Virus: Recent Emergence of Distinct Sub-lineages of the Dominant Genotype 1
Source: PLoS Negl Trop Dis. 2015 Nov 24;9(11):e0004240. doi: 10.1371/journal.pntd.0004240 (PMC4657991; doi:10.1371/journal.pntd.0004240)
Supplement: S4 Table — (DOCX) [file pntd.0004240.s004.docx]

**S4 Table. Unique amino acids in the pre-membrane and envelope protein sequences of MVEV that define genotype or sub-lineage.**

| Site^a^ | Genotype or sub-lineage | | | | | Location | Substitution type |
| --- | --- | --- | --- | --- | --- | --- | --- |
|  | G1A | G1B | G2 | G3 | G4 |  |  |
| prM |  | | | | | | |
| 24 | A | **V** | A | A | A | c strand | Conservative |
| 77 | N | N | N | **H** | N | g strand | Non-conservative |
| Env |  | | | | | | |
| 15 | A | A | **V** | A | A | DI A_O_-B_O_ loop | Conservative |
| 21 | V | V | **I** | V | V | DI A_O_-B_O_ loop | Conservative |
| 55 | L | L | **V** | L | L | DII a strand | Conservative |
| 64 | T | T | T | T | **I** | DII a-b loop | Non-conservative |
| 72 | S | S | **A** | S | S | DII b strand | Non-conservative |
| 126 | A | A | **T** | A | A | DII e strand | Non-conservative |
| 157 | T | T | **S** | T | T | DI αA helix | Conservative |
| 165 | **V** | **V** | A | A | A | DI F_O_ strand | Conservative |
| 180 | **L** | M | M | M | M | DI G_O_-H_O_ loop | Conservative |
| 187 | T | T | T | **A** | T | DI H_O_ strand | Non-conservative |
| 205 | T | T | **S** | T | T | DII f strand | Conservative |
| 229 | **A** | **A** | S | S | S | DII h loop | Non-conservative |
| 232 | E | E | **D** | E | E | DII i loop | Conservative |
| 240 | **V** | **V** | M | M | M | DII i loop | Conservative |
| 270 | I | I | I | **V** | I | DII k loop | Conservative |
| 275 | S | P | **T** | A | A | DII k loop  (DI-DII hinge) | Both types |
| 276 | S | S | **G** | S | S | DII k loop  (DI-DII hinge) | Non-conservative |
| 330 | T | T | **A** | T | T | DIII B-C loop | Non-conservative |
| 352 | V | V | **I** | V | V | DIII D loop | Conservative |
| 369 | A | A | **S** | A | A | DIII D loop | Non-conservative |
| 442 | V | V | V | **I** | V | Stem EH2 domain | Conservative |
| 461 | S | S | **T** | S | S | Transmembrane domain | Conservative |
| 474 | V | V | **I** | V | V | Transmembrane domain | Conservative |

^a^Amino acid symbols in bold font with grey shading are unique to the corresponding genotype.
