# Supplementary material for: The Molecular Epidemiology and Evolution of Murray Valley Encephalitis Virus: Recent Emergence of Distinct Sub-lineages of the Dominant Genotype 1
Source: PLoS Negl Trop Dis. 2015 Nov 24;9(11):e0004240. doi: 10.1371/journal.pntd.0004240 (PMC4657991; doi:10.1371/journal.pntd.0004240)
Supplement: S5 Table — (DOCX) [file pntd.0004240.s005.docx]

**S5 Table. Evidence for positive and negative selection in the prM and Env genes (668 amino acids) of Murray Valley encephalitis using six different methods implemented in the DataMonkey server of the HyPhy software package.**

| Selection Method | Selection | |
| --- | --- | --- |
|  | Positive | Negative |
| SLAC^a^ | 0 | 82 |
| FEL^a^ | 1 | 159 |
| IFEL^a^ | 1 | 115 |
| REL^b^ | 1 | 9 |
| FUBAR^c^ | 1 | 499 |
| MEME^d^ | 1 | NA |

^a^Number of sites where *P*< 0.05.

^b^Number of sites where Bayes factor >100.

^c^Number of sites where posterior probability (β>α) >0.9.

^d^Number of sites where *P*<0.01.
