# Supplementary material for: The Molecular Epidemiology and Evolution of Murray Valley Encephalitis Virus: Recent Emergence of Distinct Sub-lineages of the Dominant Genotype 1
Source: PLoS Negl Trop Dis. 2015 Nov 24;9(11):e0004240. doi: 10.1371/journal.pntd.0004240 (PMC4657991; doi:10.1371/journal.pntd.0004240)
Supplement: S6 Table — (DOCX) [file pntd.0004240.s006.docx]

**S6 Table. Murray Valley encephalitis virus strains encoding the S332G amino acid substitution in the envelope protein.**

| Strain | Genotype | Year | Location | Mosquito species isolated from |
| --- | --- | --- | --- | --- |
| OR1109 | 1 | 1977 | WA | *Culex annulirostris* |
| PH491 | 1 | 1981 | WA | *Cx. annulirostris* |
| AN505 | 1 | 1984 | WA | *Aedes normanensis* |
| K16383 | 1 | 1994 | WA | *Cx. annulirostris* |
| K16825 | 1 | 1994 | WA | *Cx. annulirostris* |
| K41994 | 1B | 2000 | WA | *Cx. annulirostris* |
| P6461 | 1B | 2000 | WA | *Cx. annulirostris* |
| K47457 | 1B | 2001 | WA | *Cx. annulirostris* |
| 2001341 | 1B | 2001 | QLD | *Cx. annulirostris* |
| K49901 | 1B | 2002 | WA | *Cx. annulirostris* |
| K49077 | 1B | 2002 | WA | *Cx. annulirostris* |
| K49926 | 1B | 2002 | WA | *Cx. annulirostris* |
| K50609 | 1 | 2003 | WA | *Ae. normanensis* |
| K70310 | 1A | 2009 | WA | *Cx. annulirostris* |
