# Supplementary material for: The Molecular Epidemiology and Evolution of Murray Valley Encephalitis Virus: Recent Emergence of Distinct Sub-lineages of the Dominant Genotype 1
Source: PLoS Negl Trop Dis. 2015 Nov 24;9(11):e0004240. doi: 10.1371/journal.pntd.0004240 (PMC4657991; doi:10.1371/journal.pntd.0004240)
Supplement: S7 Table — Amino acids corresponding to genotype-defining residues are indicated by (*). (DOCX) [file pntd.0004240.s007.docx]

**S7 Table. Amino acid sites within the prM and Env proteins under directional selection.** Amino acids corresponding to genotype-defining residues are indicated by (*).

| Site | Composition^a^ | Location^b^ | MRCA residue^c^ | Inferred substitutions^d^ | Target | DEPS EBF^e^ | Genotype of target | Substitution type |
| --- | --- | --- | --- | --- | --- | --- | --- | --- |
| prM |  |  |  |  |  |  |  |  |
| 24* | A_26_V_20_ | c strand | A | A_0_↔_20_V | V | V:>10^5^ | G1B | Conservative |
| Env |  |  |  |  |  |  |  |  |
| 7 | S_41_G_5_ | DI N-terminus | S | G_5_↔_0_S | G | G: 325.2 | G2 | Non-conservative |
| 15* | A_40_V_6_ | DI A_O_-B_O_ loop | A | A_0_↔_6_V | V | V: 851.0 | G2 | Conservative |
| 55* | L_40_V_6_ | DII a strand | L | L_0_↔_6_V | V | V:7874.0 | G2 | Conservative |
| 72* | S_40_A_6_ | DII b strand | S | A_6_↔_0_S | A | A:1088.1 | G2 | Non-conservative |
| 123 | N_37_S_9_ | DII e strand | N | N_0_↔_9_S | S | S:>10^5^ | G1B^f^, G2-4 | Conservative |
| 126* | A_39_T_6_V_1_ | DII e strand | A | A_0_↔_6_T A_0_↔_1_V | T | T: 584.1 | G2 | Non-conservative |
| 165* | V_38_A_8_ | DI F_O_ strand | V | A_8_↔_0_V | A | A:>10^5^ | G2-4 | Conservative(Abe, Kuzuhara et al. 2003) |
| 229* | A_38_S_8_ | DII h loop | A | A_0_↔_8_S | S | S:8752.1 | G2-4 | Non-conservative |
| 238 | V_35_I_11_ | DII i loop | V | I_10_↔_1_V | I | I: 409.1 | G1, G3-4 | Conservative |
| 240* | V_38_M_8_ | DII i loop | V | M_8_↔_0_V | M | M:>10^5^ | G2-4 | Conservative |
| 275* | P_23_S_15_T_6_A_2_ | DII k loop  (DI-DII hinge: αB-I strands) | P | A_2_↔_0_P P_2_↔_13_S P_0_↔_5_T S_0_↔_1_T | S/T | S:>10^5^ T:>10^5^ | G1  G2 | Non-conservative  Non-conservative |
| 276* | S_40_G_6_ | DII k loop  (DI-DII hinge: αB-I strands) | S | G_6_↔_0_S | G | G:3991.9 | G2 | Non-conservative |
| 307 | E_41_G_5_ | DII-DIII hinge (I_O_-A strands) | E | E_0_↔_5_G | G | G: 426.3 | G1B | Non-conservative |
| 330* | T_40_A_6_ | DIII B-C loop | T | A_6_↔_0_T | A | A: 279.5 | G2 | Non-conservative |
| 332 | S_37_G_9_ | DIII B-C loop | S | G_9_↔_0_S | G | G:>10^5^ | G1 | Non-conservative |
| 369* | A_39_S_6_V_1_ | DIII loop D | A | A_0_↔_6_S A_0_↔_1_V | S | S: 263.9 | G2 | Non-conservative |
| 461* | S_40_T_6_ | Transmembrane | S | S_0_↔_6_T | T | T: 561.9 | G2 | Conservative |

^a^Amino acid site under directional selection.

^b^Based on the crystal structures of the West Nile virus pr (PDB accession no. 3C5X; Li et al., 2008) and envelope proteins (PDB accession no. 2HG0; Nybakken et al., 2006).

^c^Reconstructed Most Recent Common Ancestor (MRCA) at site.

^d^Amino acid substitutions inferred where H_n_↔_m_K indicates *n* substitutions from H to K, and *m* substitutions from K to H.

^e^Empirical Bayes Factor for evidence in favour of a directional selection model at the site for the target residue. DEPS, directional evolution in protein sequences.

^f^Encoded by only a single strain of G1 (K41994), belonging to G1A.
