# Supplementary material for: The Molecular Epidemiology and Evolution of Murray Valley Encephalitis Virus: Recent Emergence of Distinct Sub-lineages of the Dominant Genotype 1
Source: PLoS Negl Trop Dis. 2015 Nov 24;9(11):e0004240. doi: 10.1371/journal.pntd.0004240 (PMC4657991; doi:10.1371/journal.pntd.0004240)
Supplement: S8 Table — (DOCX) [file pntd.0004240.s008.docx]

**S8 Table. Virulence of Murray Valley encephalitis virus strains, representing genotypes 1 and 2, in 18-day old Swiss mice following intraperitoneal inoculation.**

| Virus (genotype) | Dose  (Log_10_ TCID_50_) | Mortality (%) | AST^a^  (days) | HD_50_^c^  (Log_10_ TCID_50_) |
| --- | --- | --- | --- | --- |
| MVE-1-51 (G1) | 0 | 0/6 (0%) | N/A | 0.88 |
|  | 1 | 4/6 (66.7%) | 6.3 |  |
|  | 2 | 5/6 (83.3%) | 5.2 |  |
| OR156 (G2) | 2 | 1/6 (16.7%) | 12.0**^b^** | >4.00 |
|  | 3 | 0/6 (0%) | N/A |  |
|  | 4 | 0/6 (0%) | N/A |  |
| K59532 (G2) | 2 | 2/6 (33.3%) | 10.0 | >4.00 |
|  | 3 | 1/6 (16.7%) | 10.0**^b^** |  |
|  | 4 | 0/6 (0%) | N/A |  |

^a^Average survival time.

**^b^**Survival time of mice that died or were euthanised.

^c^50% humane end point dose.

**Methods**

Groups of six 18-day old Swiss ARC mice (Animal Resources Centre, Murdoch, Western Australia, Australia) were injected by the intraperitoneal (IP) route with 50µl of a range of 10-fold serial dilutions of virus in PBS. A group of six control mice were inoculated with 50µl of sterile PBS. Mice were housed in clean individually ventilated cages and were provided with water and food ad libitum. Mice were weighed every morning to monitor for signs of anorexia. Infected mice were monitored twice daily until the first signs of the disease were observed, after which, they were examined every two hours. Mice that showed signs of disease (hunching, lethargy, eye closure, convulsion, change of skin colour, hind leg flaccid paralysis) or that lost 15% of their pre-inoculation body weight were euthanised by IP injection of a lethal dose of pentobarbitol sodium (150 mg/kg body weight) followed by cervical dislocation. After 21 days, surviving mice were euthanised as above. To reduce distress to experimental animals, the HD_50_ method was used, which does not significantly differ from LD_50_ [1]. The HD_50_ was calculated for each virus using the method of Reed and Muench [2]. The AST was also determined for each infection group by calculating the mean time to death (in days) for mice that succumbed to infection.

**References**

1. Wright A, Phillpotts R (1998) Humane endpoints are an objective measure of morbidity in Venezuelan encephalomyelitis virus infection of mice. Arch Virol 143: 155-1162.

2. Reed L, Muench H (1938) A simple method of estimating fifty per cent endpoints. Am J Hyg 27: 493-497.
