# Supplementary material for: The Molecular Epidemiology and Evolution of Murray Valley Encephalitis Virus: Recent Emergence of Distinct Sub-lineages of the Dominant Genotype 1
Source: PLoS Negl Trop Dis. 2015 Nov 24;9(11):e0004240. doi: 10.1371/journal.pntd.0004240 (PMC4657991; doi:10.1371/journal.pntd.0004240)
Supplement: S1 Fig — NJ trees (A, C and E) were estimated with the maximum composite likelihood model with a gamma distribution. ML trees (B, D and F) were estimated using a general time-reversible model of nucleotide substitution with a gamma distribution and invariant sites. Numbers at the nodes represent bootstrap support as a percentage of 1000 replicates; only values ≥50% are shown. The scale bar indicates nucleotide substitutions per site. Each tree was rooted with the analogous sequence of JEV, however this has been removed to improve visual resolution of the tree. (PDF) [file pntd.0004240.s009.pdf]

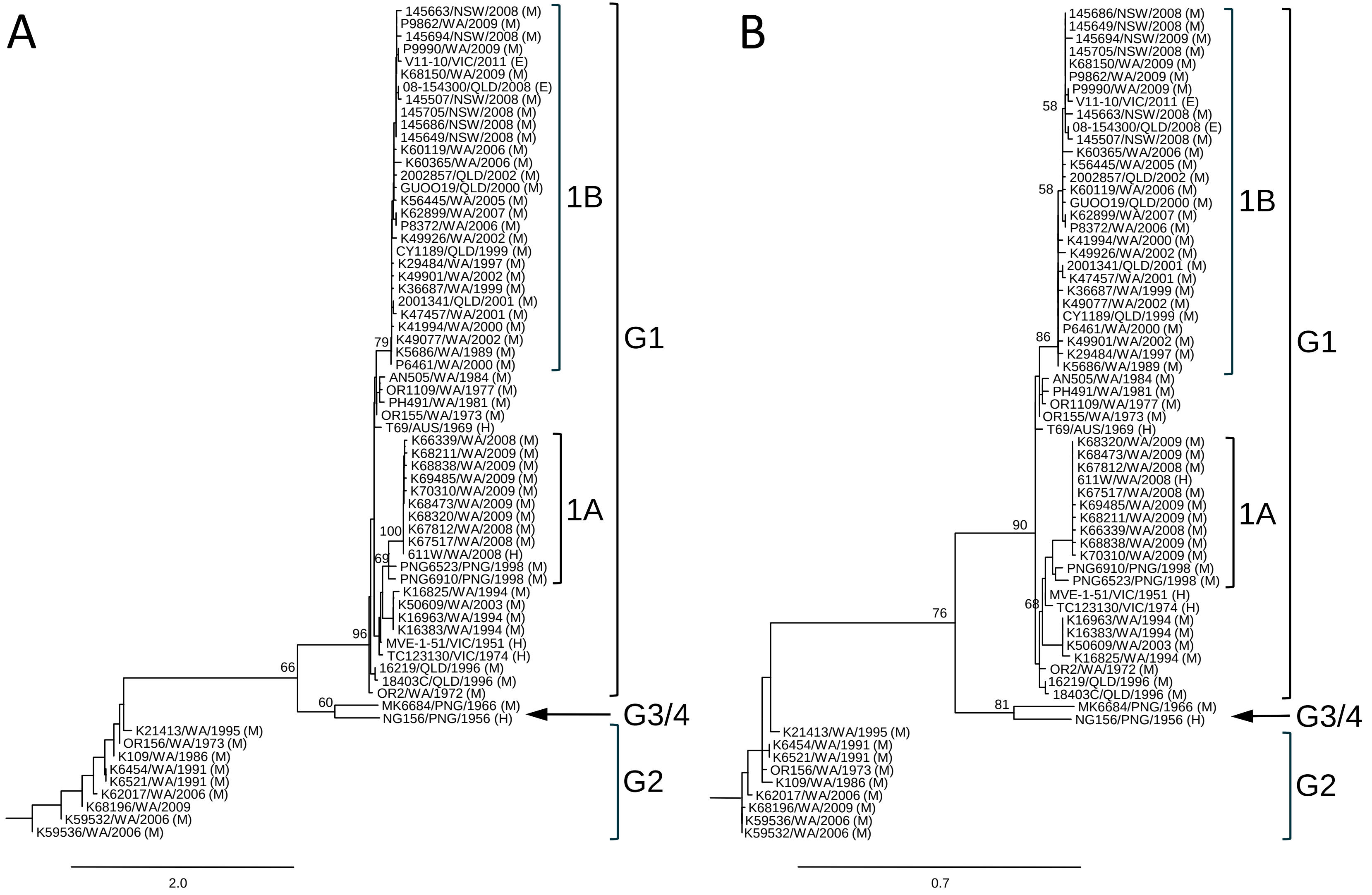
A
145663/NSW/2008 (M)
P9862/WA/2009 (M)
145694/NSW/2008 (M)
P9990/WA/2009 (M)
V11-10/VIC/2011 (E)
K68150/WA/2009 (M)
08-154300/QLD/2008 (E)
145507/NSW/2008 (M)
145705/NSW/2008 (M)
145686/NSW/2008 (M)
145649/NSW/2008 (M)
K60119/WA/2006 (M)
K60365/WA/2006 (M)
2002857/QLD/2002 (M)
GUOO19/QLD/2000 (M)
K56445/WA/2005 (M)
K62899/WA/2007 (M)
P8372/WA/2006 (M)
K49926/WA/2002 (M)
CY1189/QLD/1999 (M)
K29484/WA/1997 (M)
K49901/WA/2002 (M)
K36687/WA/1999 (M)
2001341/QLD/2001 (M)
K47457/WA/2001 (M)
K41994/WA/2000 (M)
K49077/WA/2002 (M)
K5686/WA/1989 (M)
P6461/WA/2000 (M)
79
AN505/WA/1984 (M)
OR1109/WA/1977 (M)
PH491/WA/1981 (M)
OR155/WA/1973 (M)
T69/AUS/1969 (H)
K66339/WA/2008 (M)
K68211/WA/2009 (M)
K68838/WA/2009 (M)
K69485/WA/2009 (M)
K70310/WA/2009 (M)
K68473/WA/2009 (M)
K68320/WA/2009 (M)
K67812/WA/2008 (M)
K67517/WA/2008 (M)
611W/WA/2008 (H)
100
69
PNG6523/PNG/1998 (M)
PNG6910/PNG/1998 (M)
K16825/WA/1994 (M)
K50609/WA/2003 (M)
K16963/WA/1994 (M)
K16383/WA/1994 (M)
MVE-1-51/VIC/1951 (H)
TC123130/VIC/1974 (H)
96
66
16219/QLD/1996 (M)
18403C/QLD/1996 (M)
OR2/WA/1972 (M)
60
MK6684/PNG/1966 (M)
NG156/PNG/1956 (H)
K21413/WA/1995 (M)
OR156/WA/1973 (M)
K109/WA/1986 (M)
K6454/WA/1991 (M)
K6521/WA/1991 (M)
K62017/WA/2006 (M)
K68196/WA/2009
K59532/WA/2006 (M)
K59536/WA/2006 (M)
1B
1A
G1
G3/4
G2
2.0
B
145686/NSW/2008 (M)
145649/NSW/2008 (M)
145694/NSW/2009 (M)
145705/NSW/2008 (M)
K68150/WA/2009 (M)
P9862/WA/2009 (M)
P9990/WA/2009 (M)
V11-10/VIC/2011 (E)
58
145663/NSW/2008 (M)
08-154300/QLD/2008 (E)
145507/NSW/2008 (M)
K60365/WA/2006 (M)
K56445/WA/2005 (M)
2002857/QLD/2002 (M)
58
K60119/WA/2006 (M)
GUOO19/QLD/2000 (M)
K62899/WA/2007 (M)
P8372/WA/2006 (M)
K41994/WA/2000 (M)
K49926/WA/2002 (M)
2001341/QLD/2001 (M)
K47457/WA/2001 (M)
K36687/WA/1999 (M)
K49077/WA/2002 (M)
CY1189/QLD/1999 (M)
P6461/WA/2000 (M)
86
K49901/WA/2002 (M)
K29484/WA/1997 (M)
K5686/WA/1989 (M)
AN505/WA/1984 (M)
PH491/WA/1981 (M)
OR1109/WA/1977 (M)
OR155/WA/1973 (M)
T69/AUS/1969 (H)
K68320/WA/2009 (M)
K68473/WA/2009 (M)
K67812/WA/2008 (M)
611W/WA/2008 (H)
K67517/WA/2008 (M)
K69485/WA/2009 (M)
K68211/WA/2009 (M)
K66339/WA/2008 (M)
K68838/WA/2009 (M)
K70310/WA/2009 (M)
90
PNG6910/PNG/1998 (M)
PNG6523/PNG/1998 (M)
MVE-1-51/VIC/1951 (H)
68
TC123130/VIC/1974 (H)
76
K16963/WA/1994 (M)
K16383/WA/1994 (M)
K50609/WA/2003 (M)
K16825/WA/1994 (M)
OR2/WA/1972 (M)
16219/QLD/1996 (M)
18403C/QLD/1996 (M)
81
MK6684/PNG/1966 (M)
NG156/PNG/1956 (H)
K21413/WA/1995 (M)
K6454/WA/1991 (M)
K6521/WA/1991 (M)
OR156/WA/1973 (M)
K109/WA/1986 (M)
K62017/WA/2006 (M)
K68196/WA/2009 (M)
K59536/WA/2006 (M)
K59532/WA/2006 (M)
1B
1A
G1
G3/4
G2
0.7

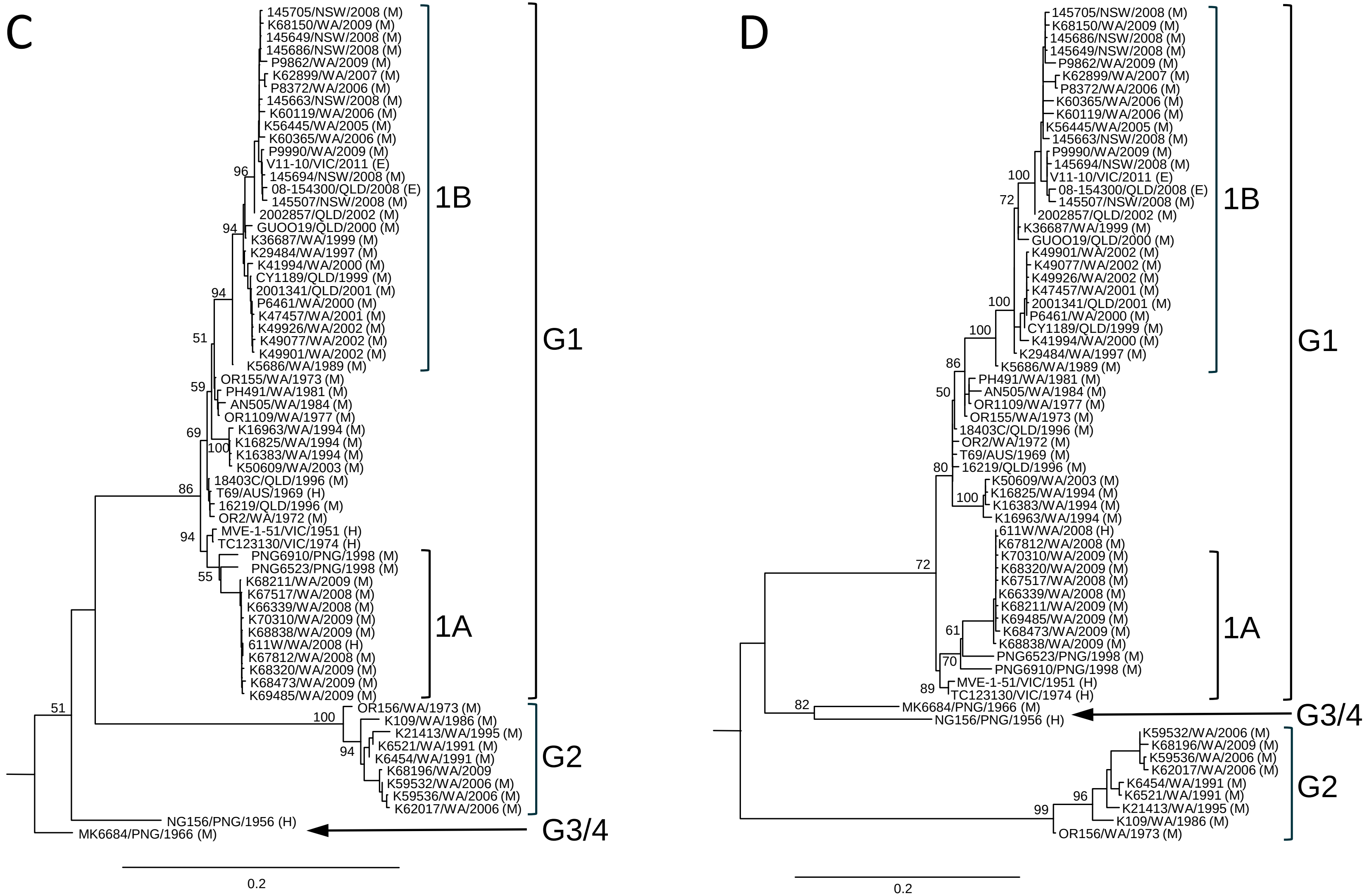
C
145705/NSW/2008 (M)
K68150/WA/2009 (M)
145649/NSW/2008 (M)
145686/NSW/2008 (M)
P9862/WA/2009 (M)
K62899/WA/2007 (M)
P8372/WA/2006 (M)
145663/NSW/2008 (M)
K60119/WA/2006 (M)
K56445/WA/2005 (M)
K60365/WA/2006 (M)
P9990/WA/2009 (M)
V11-10/VIC/2011 (E)
145694/NSW/2008 (M)
08-154300/QLD/2008 (E)
145507/NSW/2008 (M)
2002857/QLD/2002 (M)
GUOO19/QLD/2000 (M)
K36687/WA/1999 (M)
K29484/WA/1997 (M)
K41994/WA/2000 (M)
CY1189/QLD/1999 (M)
2001341/QLD/2001 (M)
P6461/WA/2000 (M)
K47457/WA/2001 (M)
K49926/WA/2002 (M)
K49077/WA/2002 (M)
K49901/WA/2002 (M)
K5686/WA/1989 (M)
OR155/WA/1973 (M)
PH491/WA/1981 (M)
AN505/WA/1984 (M)
OR1109/WA/1977 (M)
K16963/WA/1994 (M)
K16825/WA/1994 (M)
K16383/WA/1994 (M)
K50609/WA/2003 (M)
18403C/QLD/1996 (M)
T69/AUS/1969 (H)
16219/QLD/1996 (M)
OR2/WA/1972 (M)
MVE-1-51/VIC/1951 (H)
TC123130/VIC/1974 (H)
PNG6910/PNG/1998 (M)
PNG6523/PNG/1998 (M)
K68211/WA/2009 (M)
K67517/WA/2008 (M)
K66339/WA/2008 (M)
K70310/WA/2009 (M)
K68838/WA/2009 (M)
611W/WA/2008 (H)
K67812/WA/2008 (M)
K68320/WA/2009 (M)
K68473/WA/2009 (M)
K69485/WA/2009 (M)
OR156/WA/1973 (M)
K109/WA/1986 (M)
K21413/WA/1995 (M)
K6521/WA/1991 (M)
K6454/WA/1991 (M)
K68196/WA/2009
K59532/WA/2006 (M)
K59536/WA/2006 (M)
K62017/WA/2006 (M)
NG156/PNG/1956 (H)
MK6684/PNG/1966 (M)
96
94
94
51
59
69
100
86
94
55
100
94
51
1B
1A
G1
G2
G3/4
0.2
D
145705/NSW/2008 (M)
K68150/WA/2009 (M)
145686/NSW/2008 (M)
145649/NSW/2008 (M)
P9862/WA/2009 (M)
K62899/WA/2007 (M)
P8372/WA/2006 (M)
K60365/WA/2006 (M)
K60119/WA/2006 (M)
K56445/WA/2005 (M)
145663/NSW/2008 (M)
P9990/WA/2009 (M)
145694/NSW/2008 (M)
V11-10/VIC/2011 (E)
08-154300/QLD/2008 (E)
145507/NSW/2008 (M)
2002857/QLD/2002 (M)
K36687/WA/1999 (M)
GUOO19/QLD/2000 (M)
K49901/WA/2002 (M)
K49077/WA/2002 (M)
K49926/WA/2002 (M)
K47457/WA/2001 (M)
2001341/QLD/2001 (M)
P6461/WA/2000 (M)
CY1189/QLD/1999 (M)
K41994/WA/2000 (M)
K29484/WA/1997 (M)
K5686/WA/1989 (M)
PH491/WA/1981 (M)
AN505/WA/1984 (M)
OR1109/WA/1977 (M)
OR155/WA/1973 (M)
18403C/QLD/1996 (M)
OR2/WA/1972 (M)
T69/AUS/1969 (M)
16219/QLD/1996 (M)
K50609/WA/2003 (M)
K16825/WA/1994 (M)
K16383/WA/1994 (M)
K16963/WA/1994 (M)
611W/WA/2008 (H)
K67812/WA/2008 (M)
K70310/WA/2009 (M)
K68320/WA/2009 (M)
K67517/WA/2008 (M)
K66339/WA/2008 (M)
K68211/WA/2009 (M)
K69485/WA/2009 (M)
K68473/WA/2009 (M)
K68838/WA/2009 (M)
PNG6523/PNG/1998 (M)
PNG6910/PNG/1998 (M)
MVE-1-51/VIC/1951 (H)
TC123130/VIC/1974 (H)
MK6684/PNG/1966 (M)
NG156/PNG/1956 (H)
K59532/WA/2006 (M)
K68196/WA/2009 (M)
K59536/WA/2006 (M)
K62017/WA/2006 (M)
K6454/WA/1991 (M)
K6521/WA/1991 (M)
K21413/WA/1995 (M)
K109/WA/1986 (M)
OR156/WA/1973 (M)
100
72
100
100
86
50
80
100
72
61
70
89
82
96
99
1B
1A
G1
G3/4
G2
0.2

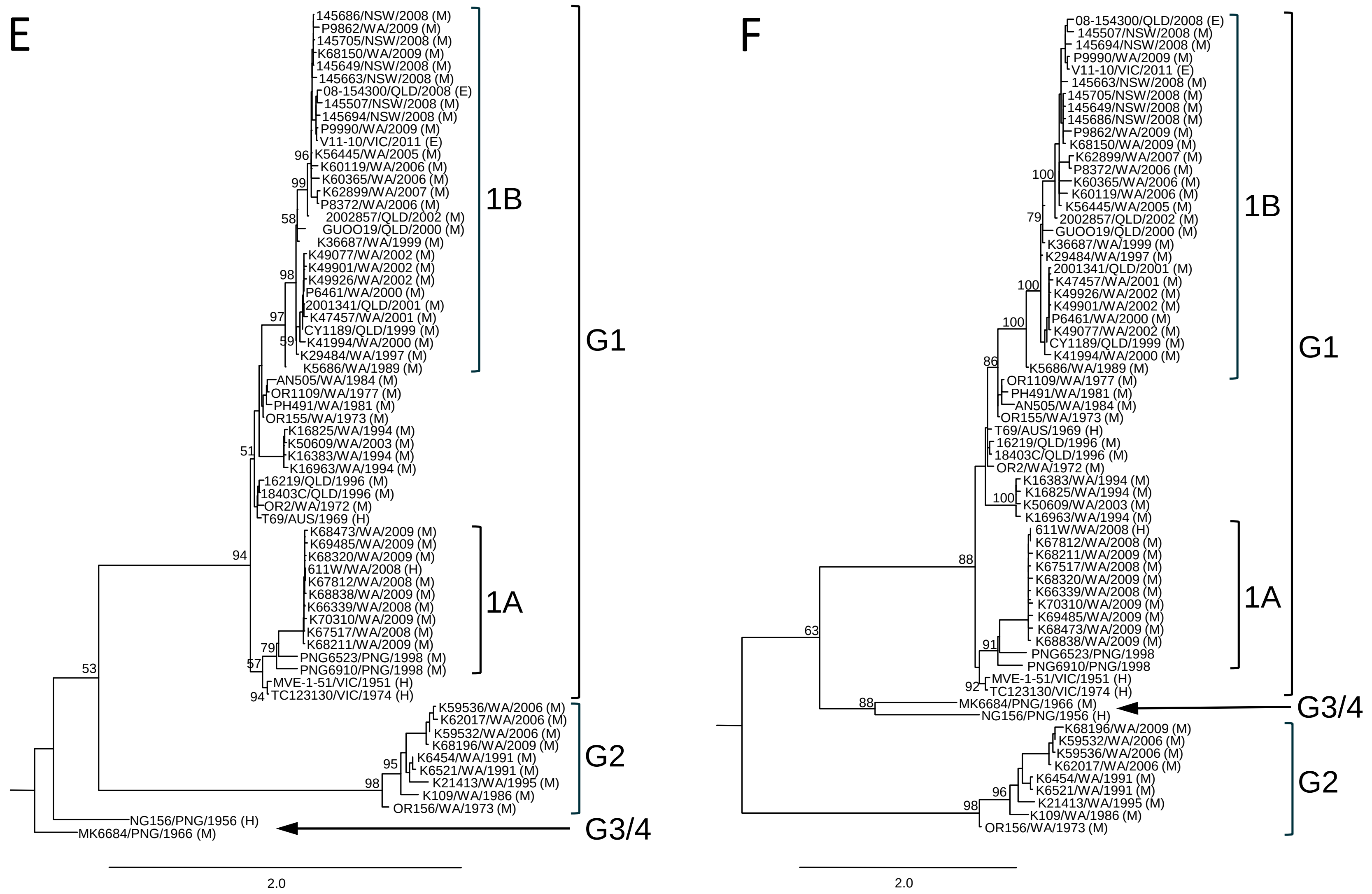

E
145686/NSW/2008 (M)
P9862/WA/2009 (M)
145705/NSW/2008 (M)
K68150/WA/2009 (M)
145649/NSW/2008 (M)
145663/NSW/2008 (M)
08-154300/QLD/2008 (E)
145507/NSW/2008 (M)
145694/NSW/2008 (M)
P9990/WA/2009 (M)
V11-10/VIC/2011 (E)
96
K56445/WA/2005 (M)
K60119/WA/2006 (M)
99
K60365/WA/2006 (M)
K62899/WA/2007 (M)
P8372/WA/2006 (M)
58
2002857/QLD/2002 (M)
GUOO19/QLD/2000 (M)
K36687/WA/1999 (M)
K49077/WA/2002 (M)
98
K49901/WA/2002 (M)
K49926/WA/2002 (M)
P6461/WA/2000 (M)
2001341/QLD/2001 (M)
97
K47457/WA/2001 (M)
CY1189/QLD/1999 (M)
59
K41994/WA/2000 (M)
K29484/WA/1997 (M)
K5686/WA/1989 (M)
1B
AN505/WA/1984 (M)
OR1109/WA/1977 (M)
PH491/WA/1981 (M)
OR155/WA/1973 (M)
K16825/WA/1994 (M)
K50609/WA/2003 (M)
51
K16383/WA/1994 (M)
K16963/WA/1994 (M)
16219/QLD/1996 (M)
18403C/QLD/1996 (M)
OR2/WA/1972 (M)
T69/AUS/1969 (H)
K68473/WA/2009 (M)
94
K69485/WA/2009 (M)
K68320/WA/2009 (M)
611W/WA/2008 (H)
K67812/WA/2008 (M)
K68838/WA/2009 (M)
K66339/WA/2008 (M)
K70310/WA/2009 (M)
K67517/WA/2008 (M)
K68211/WA/2009 (M)
79
PNG6523/PNG/1998 (M)
57
PNG6910/PNG/1998 (M)
1A
MVE-1-51/VIC/1951 (H)
94
TC123130/VIC/1974 (H)
53
G1
K59536/WA/2006 (M)
K62017/WA/2006 (M)
K59532/WA/2006 (M)
K68196/WA/2009 (M)
K6454/WA/1991 (M)
95
K6521/WA/1991 (M)
98
K21413/WA/1995 (M)
K109/WA/1986 (M)
OR156/WA/1973 (M)
G2
NG156/PNG/1956 (H)
MK6684/PNG/1966 (M)
G3/4
2.0
F
08-154300/QLD/2008 (E)
145507/NSW/2008 (M)
145694/NSW/2008 (M)
P9990/WA/2009 (M)
V11-10/VIC/2011 (E)
145663/NSW/2008 (M)
145705/NSW/2008 (M)
145649/NSW/2008 (M)
145686/NSW/2008 (M)
P9862/WA/2009 (M)
K68150/WA/2009 (M)
K62899/WA/2007 (M)
100
P8372/WA/2006 (M)
K60365/WA/2006 (M)
K60119/WA/2006 (M)
K56445/WA/2005 (M)
79
2002857/QLD/2002 (M)
GUOO19/QLD/2000 (M)
K36687/WA/1999 (M)
K29484/WA/1997 (M)
2001341/QLD/2001 (M)
100
K47457/WA/2001 (M)
K49926/WA/2002 (M)
K49901/WA/2002 (M)
100
P6461/WA/2000 (M)
K49077/WA/2002 (M)
CY1189/QLD/1999 (M)
K41994/WA/2000 (M)
86
K5686/WA/1989 (M)
1B
OR1109/WA/1977 (M)
PH491/WA/1981 (M)
AN505/WA/1984 (M)
OR155/WA/1973 (M)
T69/AUS/1969 (H)
16219/QLD/1996 (M)
18403C/QLD/1996 (M)
OR2/WA/1972 (M)
K16383/WA/1994 (M)
K16825/WA/1994 (M)
100
K50609/WA/2003 (M)
K16963/WA/1994 (M)
611W/WA/2008 (H)
K67812/WA/2008 (M)
88
K68211/WA/2009 (M)
K67517/WA/2008 (M)
K68320/WA/2009 (M)
K66339/WA/2008 (M)
63
K70310/WA/2009 (M)
K69485/WA/2009 (M)
K68473/WA/2009 (M)
K68838/WA/2009 (M)
91
PNG6523/PNG/1998
PNG6910/PNG/1998
1A
92
MVE-1-51/VIC/1951 (H)
TC123130/VIC/1974 (H)
G1
88
MK6684/PNG/1966 (M)
NG156/PNG/1956 (H)
G3/4
K68196/WA/2009 (M)
K59532/WA/2006 (M)
K59536/WA/2006 (M)
K62017/WA/2006 (M)
K6454/WA/1991 (M)
K6521/WA/1991 (M)
96
K21413/WA/1995 (M)
98
K109/WA/1986 (M)
OR156/WA/1973 (M)
G2
2.0

**S1 Fig. Phylogenetic analysis of MVEV using prM (A, B), Env (C, D) and prM-Env (E, F) gene sequences.** NJ trees (A, C and E) were estimated with the maximum composite likelihood model with a gamma distribution. ML trees (B, D and F) were estimated using a general time-reversible model of nucleotide substitution with a gamma distribution and invariant sites. Numbers at the nodes represent bootstrap support as a percentage of 1000 replicates; only values ≥50% are shown. The scale bar indicates nucleotide substitutions per site. Each tree was rooted with the analogous sequence of JEV, however this has been removed to improve visual resolution of the tree.
