# Supplementary material for: The Molecular Epidemiology and Evolution of Murray Valley Encephalitis Virus: Recent Emergence of Distinct Sub-lineages of the Dominant Genotype 1
Source: PLoS Negl Trop Dis. 2015 Nov 24;9(11):e0004240. doi: 10.1371/journal.pntd.0004240 (PMC4657991; doi:10.1371/journal.pntd.0004240)
Supplement: S2 Fig — Identical amino acids are colour-shaded. The symbols below the alignments indicate identical amino acids (*), strongly conserved (:), and weakly conserved (.) amino acids. (PDF) [file pntd.0004240.s010.pdf]

A

```
MVE-1-51/VIC/1951 (G1)   PASTE
K66339/WA/2008 (G1A)     PASTE
P8372/WA/2006 (G1B)      PASTE
K60365/WA/2006 (G1B)     PASSE
OR156/WA/1973 (G2)       PSNTD
K6521/WA/1991 (G2)       PSSTD
NG156/PNG/1956 (G3)      PSSTE
MK6684/PNG/1966 (G4)     PSSTE
                         *:.*:
```

B

```
MVE-1-51/VIC/1951 (G1)   SSST
K16383/WA/1994 (G1)      SSNT
K66339/WA/2008 (G1A)     SSST
P8372/WA/2006 (G1B)      PSNT
K60365/WA/2006 (G1B)     PSST
OR156/WA/1973 (G2)       TGST
K6521/WA/1991 (G2)       TGST
NG156/PNG/1956 (G3)      ASST
MK6684/PNG/1966 (G4)     ASST
                         ...*
```

S2 Fig. The hypervariable region (A) and DI-DII hinge motif (B) of the Envelope protein encoded by representative strains of Murray Valley encephalitis virus. Identical amino acids are colour-shaded. The symbols below the alignments indicate identical amino acids (*), strongly conserved (:), and weakly conserved (.) amino acids.
